# Light Quality and Sucrose-Regulated Detached Ripening of Strawberry with Possible Involvement of Abscisic Acid and Auxin Signaling

**DOI:** 10.3390/ijms24065681

**Published:** 2023-03-16

**Authors:** Leiyu Jiang, Xinpeng Chen, Xianjie Gu, Meiyi Deng, Xiaotong Li, Aiyang Zhou, Mengyue Suo, Weiliang Gao, Yuanxiu Lin, Yan Wang, Wen He, Mengyao Li, Qing Chen, Yong Zhang, Ya Luo, Xiaorong Wang, Haoru Tang, Yunting Zhang

**Affiliations:** 1 College of Horticulture, Sichuan Agricultural University, Chengdu 611130, China; 2 Mianyang Academy of Agricultural Sciences, Mianyang 621000, China; 3 Institute of Pomology and Olericulture, Sichuan Agricultural University, Chengdu 611130, China

**Keywords:** detached ripening, strawberry, light quality, sucrose

## Abstract

The regulation of detached ripening is significant for prolonging fruit shelf life. Although light quality and sucrose affecting strawberry fruit ripening have been widely reported, little information is available about how they co-regulate the strawberry detached ripening process. In this study, different light qualities (red light—RL, blue light—BL, and white light—WL) and 100 mM sucrose were applied to regulate the ripening of initial red fruits detached from the plant. The results showed RL-treated samples (RL + H_2_O, RL + 100 mM sucrose) had brighter and purer skin color with a higher L*, b*, and C* value, and promoted the ascorbic acid. Almost all light treatments significantly decreased TSS/TA (total soluble solid/titratable acid) and soluble sugar/TA ratio, which is exacerbated by the addition of sucrose. Blue or red light in combination with sucrose notably increased total phenolic content and decreased malondialdehyde (MDA) accumulation. In addition, blue or red light combined with sucrose increased abscisic acid (ABA) content and promoted ABA signaling by inducing ABA-INSENSITIVE 4 (*ABI4*) expression and inhibiting SUCROSE NONFERMENTING1-RELATED PROTEIN KINASE 2.6 (*SnRK2.6*) expression. The strawberries exposed to blue and red light significantly improved auxin (IAA) content compared to the control (0 d), whereas the addition of sucrose inhibited IAA accumulation. Moreover, sucrose treatment suppressed the AUXIN/INDOLE-3-ACETIC ACID 11 (*AUX/IAA11*) and AUXIN RESPONSE FACTOR 6 (*ARF6*) expression under different light qualities. Overall, these results indicated that RL/BL + 100 mM sucrose might promote the detached ripening of strawberries by regulating abscisic acid and auxin signaling.

## 1. Introduction

Fruit ripening is the initiation of fruit senescence, accompanied by genetically programmed and highly coordinated changes in flavor, color, texture, and aroma. Fruit can supply humans with several health-promoting benefits, so the regulatory mechanism underlying fruit ripening has been attracting great scientific interest [1,2]. Fleshy fruit is generally divided into climacteric and non-climacteric according to the patterns of respiration and ethylene production during fruit ripening. A growing body of evidence has suggested that more than one plant hormone acts during fruit ripening; however, the ripening of climacteric and non-climacteric fruit is mainly controlled by ethylene and ascorbic acid, respectively. In addition, environmental factors, nutrients, cultivation practices, etc., are also important factors to regulate ripening during fruit production [1]. 

Strawberries, a model for non-climacteric fruit, have enormous dietetic and economic value. However, one of the restrictive factors that affect its economic value is the very short senescent period and shelf life [3]. The regulation of strawberry fruit ripening to extend the shelf life is critical in agricultural output. In the current production system, strawberries are harvested at the fully mature stage, which is tremendously sensitive to mechanical injury, fungal infection, and physiological disorders, reducing the storage period. It has been demonstrated that strawberries are able to ripen after detachment at the green/white stage; however, nutritive value and quality in detached fruit become reduced in comparison with strawberries that ripen on the vine [4,5]. Hence, the study of the application of postharvest techniques to regulate strawberry detached ripening is significant. 

Manipulation of light quality is capable of improving vegetable and fruit postharvest quality [6,7]. Blue, red, and green light effectively accelerated the ripening of bananas by increasing ethylene generation and respiration rate and enhanced the accumulations of total sugars, ascorbic acid, and total phenols [8]. Blue light promoted anthocyanin accumulation, but delayed fruit ripening in purple pepper [9]. Sucrose is a pivotal component of fruit flavor and is also a signal molecule that can affect the postharvest ripening process. Exogenous sucrose contributed to fruit ripening and softening of kiwifruit by enhancing ethylene synthesis [10]. The application of sucrose in unripe strawberries detached at the white stage gave rise to the induction of ripening, which depended on ABA signaling [5].

Light quality and sucrose are environmentally friendly and safe factors to regulate the postharvest ripening of fruit. However, little information on their application for strawberry postharvest ripening is available. Here, the ripening-related physiological and molecular indicators of strawberry fruits that were subjected to white, red, and blue light and sucrose treatments after harvesting at the initial red stage were quantified, to better understand the mechanism underlying the regulation of detached strawberry fruit ripening and provide a supplementary strategy to minimize strawberry postharvest loss.

## 2. Results 

### 2.1. Skin Color

**Figure 1:**
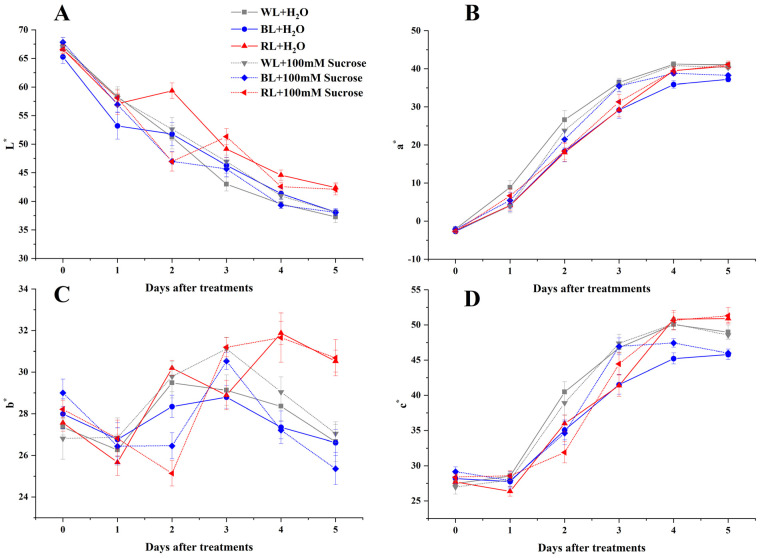
Effect of light quality and sucrose on skin color parameters of L* value (**A**), a* value (**B**), b* value (**C**), and C* value (**D**). Data represent the means ± standard error (*n* = 3).

The skin color of strawberries was described using L*, a*, b*, and C* values (Figure 1). The a* and L* values displayed an overall increasing and decreasing trend during the entire storage period, respectively (Figure 1A,B), indicating that the strawberries gradually turned red. After 5 days of storage, the L* value in RL-treated fruit (RL + H_2_O, RL + 100 mM Sucrose) was higher than other treatments; however, their a* values had no significant difference. The b* value showed a fluctuating trend in the treatment process. Eventually, the b* value of RL-treated samples was higher than the WL-treated (WL + H_2_O, WL + 100 mM Sucrose), and BL-treated (BL + H_2_O, BL + 100 mM Sucrose) samples (Figure 1C), which suggested that RL-treated fruit was more yellowish. The changing trend of the C* value was similar to the a* value. At the end of treatment, the RL-treated fruit exhibited the highest C* value, while BL-treated fruit had the lowest value. In addition, under the same light quality, with or without sucrose treatment, the value of each color parameter was very close, suggesting that sucrose treatment almost had no effect on skin color.

### 2.2. Firmness, Total Soluble Solids, Soluble Sugar, and Titratable Acid

**Figure 2:**
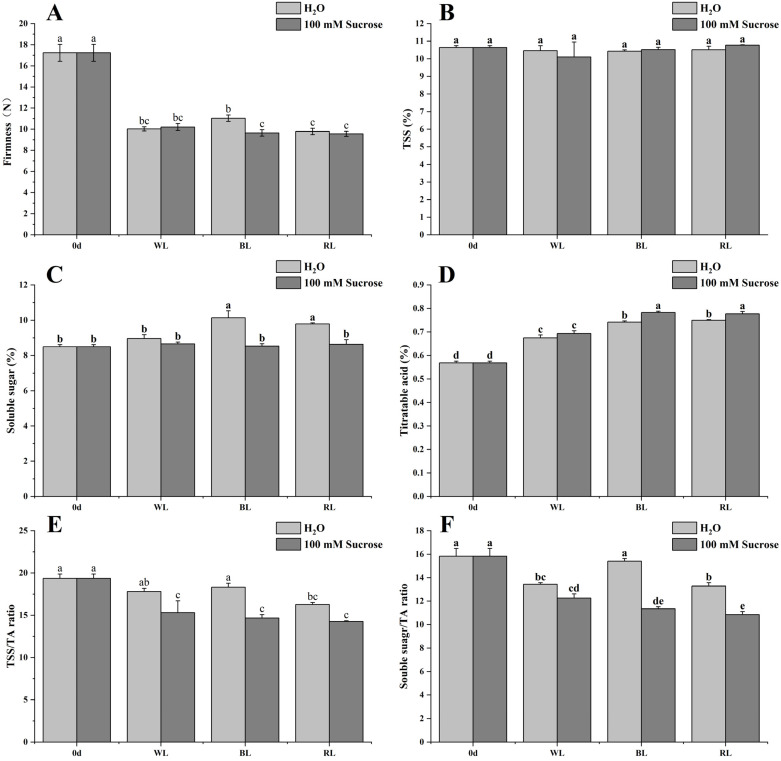
Effect of light quality and sucrose on firmness (**A**), TSS (**B**), soluble sugar (**C**), Titratable acid (**D**), TSS/TA ratio (**E**), and soluble sugar/TA ratio (**F**). Data represent the means ± standard error (*n* = 3). Different lowercase letters indicate the statistically significant difference of the mean values based on one-way analysis of variance (ANOVA) followed by Duncan’s multiple range test (*p* ≤ 0.05).

As shown in Figure 2A, the firmness of all strawberries declined sharply after 5 days of treatments. Blue light produced strawberries with higher firmness. The addition of sucrose reduced the fruit firmness under blue light but did not reduce it under white and red light. Light quality and sucrose did not change the total soluble solids (TSS) at the end of storage (Figure 2B). The soluble sugar content in fruit under blue and red light was significantly increased, and 1.27- and 1.12-fold higher compared to 0 d, while the content of soluble sugar in combination with light quality and sucrose treatment had no significant difference with 0 d (Figure 2C). There was an increase in titratable acid of fruits after 5 days of treatments. BL- (BL + H_2_O, BL + 100 mM Sucrose) and RL-treated (RL + H_2_O, RL + 100 mM Sucrose) fruit contained more titratable acid (TA) than WL-treated (WL + H_2_O, WL + 100 mM Sucrose) fruit (Figure 2D). Almost all light treatments significantly decreased the TSS/TA and soluble sugar/TA ratio, which is exacerbated by the addition of sucrose. Only blue light maintained the soluble sugar/TA ratio (Figure 2E,F).

### 2.3. Total Anthocyanins, Flavonoids, Phenolics, Ascorbic Acid, and Antioxidant Capacity

**Figure 3:**
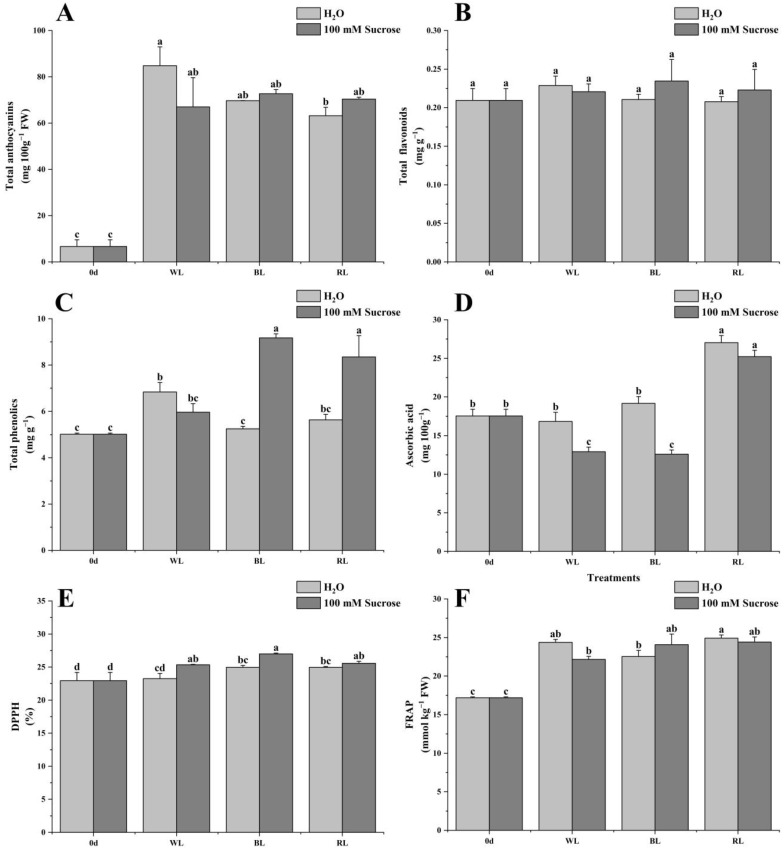
Effect of light quality and sucrose on total anthocyanins (**A**), flavonoids (**B**), phenolics (**C**), ascorbic acid (**D**), DPPH (**E**), and FRAP (**F**). Data represent the means ± standard error (*n* = 3). Different lowercase letters indicate the statistically significant difference of the mean values based on one-way analysis of variance (ANOVA) followed by Duncan’s multiple range test (*p* ≤ 0.05).

The content of total anthocyanins in treated samples was boosted significantly compared to 0 d. There was no remarkable difference among all treated samples except that anthocyanin content in red light treatment was lower than that in white light (Figure 3A). No difference was found in terms of total flavonoid content among all samples (Figure 3B). Red and blue light did not affect the total phenolics, while white light significantly increased its content. However, strawberries with BL + 100 mM Sucrose and RL + 100 mM Sucrose were higher than other treatments and exhibited 1.82-fold and 1.66-fold higher compared to 0 d in total phenolics (Figure 3C). Red light remarkably induced the accumulation of ascorbic acid; however, blue and white light had no influence on it when compared to 0 d. Ascorbic acid in RL-treated samples (RL + H_2_O, RL + 100 mM Sucrose) exhibited a higher content than other samples, while WL + 100 mM Sucrose and BL + 100 mM Sucrose treatments manifested significantly lower content than WL + H_2_O, BL + H_2_O, and 0 d samples, indicating that sucrose may negatively regulate ascorbic acid production (Figure 3D). DPPH and FRAP were used to reflect the total antioxidant capacity. Clearly, almost all treatments could elevate the fruit total antioxidant (Figure 3E,F).

### 2.4. Reactive Oxygen Species (ROS) and Malondialdehyde (MDA) Production

**Figure 4:**
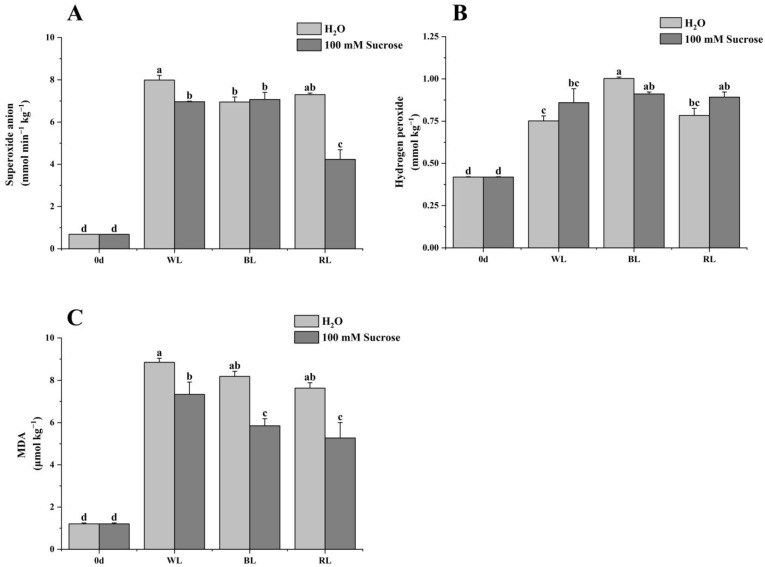
Effect of light quality and sucrose on superoxide anion (**A**), hydrogen peroxide (**B**), and MDA (**C**). Data represent the means ± standard error (*n* = 3). Different lowercase letters indicate the statistically significant difference of the mean values based on one-way analysis of variance (ANOVA) followed by Duncan’s multiple range test (*p* ≤ 0.05).

The ROS and MDA in all treated groups were dramatically increased compared to 0 d (Figure 4). The blue-light and red-light-treated groups exhibited lower levels of superoxide anion than the white-light treatment. Sucrose can significantly reduce the production of superoxide anion under red- and white-light treatments (Figure 4A). Conversely, the hydrogen peroxide content in blue light and red light was relatively higher; however, the addition of sucrose did not significantly change the hydrogen peroxide content in the respective light treatments (Figure 4B). No significant difference was observed in MDA content in strawberries treated with different light qualities. It was notably revealed that the combination of sucrose and light qualities remarkably decreased MDA content (Figure 4C). The above findings indicated that light qualities promoted strawberries’ ROS and MDA production, while sucrose had the opposite effect.

### 2.5. ABA and IAA Signaling

**Figure 5:**
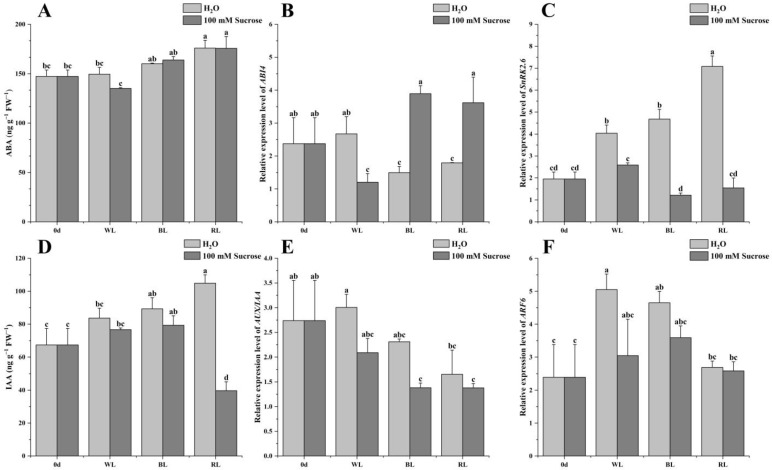
Effect of light quality and sucrose on ABA and IAA signaling. (**A**) ABA content, (**B**) *ABI4* expression level, (**C**) *SnRK2.6* expression level, (**D**) IAA content, (**E**) *AUX/IAA11* expression level, (**F**) *ARF6* expression level. Data represent the means ± standard error (*n* = 3). Different lowercase letters indicate the statistically significant difference of the mean values based on one-way analysis of variance (ANOVA) followed by Duncan’s multiple range test (*p* ≤ 0.05).

Different light qualities, especially blue and red light, increased both ABA and IAA content (Figure 5A,D). White and blue light induced the ABA accumulation; however, no significant difference was observed between them and 0 d, whereas red light played a significant role in increasing ABA content compared to 0 d and white light. Sucrose treatment did not change the light quality effect on ABA content (Figure 5A). The expression level of *ABI4* was inhibited under blue and red light, and the expression level of *SnRK2.6* was induced. Interestingly, the sucrose addition could reverse this phenomenon (Figure 5B,C). The strawberries exposed to blue and red light significantly improved IAA content compared to 0 d, whereas the addition of sucrose inhibited IAA accumulation. The IAA content in fruit under red light was significantly higher than 0 d and white light; however, it had the lowest level among all samples when sucrose was introduced (Figure 5D). The expression levels of *AUX/IAA11* and *ARF6* were significantly downregulated by red light, compared to white light. Sucrose treatment suppressed the *AUX/IAA11* and *ARF6* expression under different light qualities (Figure 5E,F).

### 2.6. Principal Component Analysis

**Figure 6:**
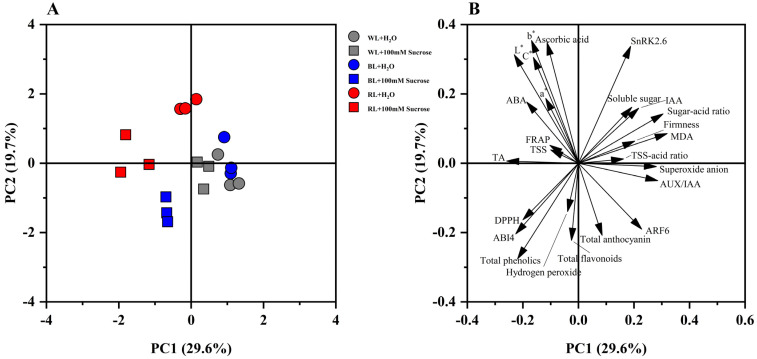
PCA analysis. (**A**) PCA score plot; (**B**) PCA loading plot.

Principal component analysis (PCA) was performed to obtain a comprehensive overview of variation in the quality indexes of strawberries subjected to different treatments. The first and second principal components (PC1 and PC2) explained 29.6% and 19.7% of the total variance, respectively. The samples subjected to RL + 100 mM sucrose and BL + 100 mM sucrose can be obviously separated from other samples along PC1 loaded in the negative direction of PC1 (Figure 6A). Additionally, the TSS, FRAP, skin color, ascorbic acid, ABA, and titratable acid were positively related to RL + 100 mM sucrose treatments. Furthermore, total flavonoids, total phenolics, hydrogen peroxide, ABI4 gene expression, and DPPH were positively related to RL + 100 mM sucrose and BL + 100 mM sucrose. Still, a performance of total anthocyanin, IAA, the expression of *AUX/IAA11*, *ARF6,* and *SnRK2.6* genes, firmness, soluble sugar, sugar–acid ratio, TSS–acid ratio, and superoxide anion were observed in WL + H_2_O, WL + 100 mM sucrose and BL+ H_2_O (Figure 6B).

## 3. Discussion

Fleshy fruits are important in the human diet due to their nutritional value. Fruit ripening is synergistically controlled by intrinsic developmental factors and various environmental cues [11]. Light, besides being an essential source of energy for driving photosynthesis, is an environmental cue that modulates many aspects of plant biology such as germination, stem elongation, flowering, and fruit ripening [9,12,13]. It has been demonstrated that light quality, especially blue and red light is an important factor of light condition which affects fruit maturity, quality, and metabolism [14]. Furthermore, sucrose, in addition to being a metabolic resource and energy supplier, has been suggested as a signal molecule to involve in the regulation of fruit ripening [15].

Fruit ripening is a complex process that has dramatic changes in color, texture, etc. In this study, different light qualities (WL, RL, and BL) promoted fruit skin coloration with an increasing a* value after 5 days of continuous irradiation during strawberry ripening; however, there was no significant difference among them, which also could be reflected from total anthocyanin content. This was consistent with a previous study on another strawberry cultivar ‘Tokun’ [16]. However, some experiments reported that pre-harvest treatments with red or blue light resulted in reddish coloration and a higher anthocyanin synthesis compared to white light [17,18]. Kadomura-Ishikawa, et al. [19] used several wavelengths of light to irradiate white-stage strawberries and found that anthocyanin content in red-light-treated fruit was significantly lower than in white and blue light. This suggested that light-quality-induced fruit coloration and pigment accumulation may be affected by cultivar, species, maturity stage, or even treatment condition. In addition, the effect of different light qualities combined with sucrose treatments almost did not differ significantly from the respective light treatments on fruit skin and anthocyanin accumulation. This was partially in agreement with the one concluded by Luo, et al. [20].

The firmness of strawberries, regardless of treatment, declined; however, compared to WL and other treatments, a higher firmness was observed in BL-treated fruit. Also in tomatoes, blue light caused the slowest softening trajectory as reported by Li, et al. [21]; however, the addition of sucrose significantly reduced the fruit firmness under blue light. The fruit TSS or total flavonoids had no significant difference under light quality and sucrose treatments. Luo, et al. [20] found that sucrose, regardless of concentration, had no significant impact on TSS and total flavonoid content in strawberries at different stages. Blue or red light increased both sugar and titratable acid content. Interestingly, sucrose combined with them had the opposite effect. The majority of studies have shown that the short-wavelength spectrum (e.g., UV-A, blue light) is more conducive to increasing ascorbic acid content compared to the long-wavelength spectrum [22,23]. However, blue-light treatment seemed not to have a remarkable effect on ascorbic acid content in strawberries [24,25,26], which was consistent with the present result. By contrast, red-light irradiation could effectively induce ascorbic acid accumulation, while sucrose addition reduced it. Additionally, the present study showed that sucrose combined with different light-quality treatments reduced the generation of ROS and MDA, indicating sucrose may contribute to inhibiting the strawberry postharvest senescence. Di, et al. [27] reported that sucrose application delayed senescence and maintained the postharvest quality of baby mustard. 

The ripening processes of fruits involve complex cross-talks among multiple plant hormones [28]. A decrease in IAA content or an increase in ABA content in the receptacle contributes to promoting strawberry fruit ripening [29,30]. It has been reported that ABA and IAA play an integrated and synergistic role to manipulate strawberry fruit ripening [31,32]. In this study, blue and red light stimulated both ABA and IAA levels, and they, in combination with sucrose, maintained the high ABA level but decreased IAA content. The expression of ABA and IAA signaling components is closely related to strawberry ripening and causes changes in fruit quality [33,34]. ABI4 and SnRK2.6 play contrasting roles in the ABA signaling pathway. ABI4 positively regulates strawberry fruit ripening, whereas SnRK2.6 plays a negative role in the ABA signaling pathway [35]. The data showed that blue or red light combined with sucrose promoted the ABA signaling pathway by increasing *ABI4* expression and decreasing *SnRK2.6* expression. In addition, the two treatments inhibited the IAA signaling by decreasing *AUX/IAA11* and *ARF6* expression.

## 4. Materials and Methods

### 4.1. Plant Material and Treatments

Strawberries (*Fragaria × ananassa* Duch, ‘Benihoppe’) at the initial red stage were obtained from a local farm in Wenjiang District, Chengdu, China. The detached fruit with 30 mm of peduncle was transported to the laboratory within two hours. After eliminating damaged and diseased ones, 105 strawberries with uniform size and maturity were selected, 15 of which were immediately stored at 80 °C for analysis of 0 d, and the remaining 90 strawberries were randomly divided into six treatments: WL (white light) +H_2_O, WL + 100 mM Sucrose, BL (blue light) + H_2_O, BL + 100 mM Sucrose, RL (red light) +H_2_O, RL + 100 mM Sucrose. Each treatment with 3 replicates of 5 fruits per replicate was immersed in a respective solution for 10 min and placed in plastic containers after drying for about 30 min at room temperature. After that, each treatment was sent to an incubator installed with white, blue (450 nm), or red (730 nm) light, respectively. All treatments were stored in the surroundings under 16 h of 100 μmol·m^−2^·s^−1^ light and 8 h of dark each day at 20 °C with (85 ± 5)% relative humidity for 5 d. The skin color of treated samples was evaluated every day.

### 4.2. Skin Color

The skin color was evaluated at two opposite points around the equator on each fruit using a chromameter (CR-400, Konica Minolta, Japan) and expressed in the CIE color space (L*, a*, b* and chroma C*), where value L* indicated the lightness from black (0) to white (100), value a* ranged from green (−60) to red (60), and value b* varied from blue (−60) to yellow (60). Chromaticity (C*) depicted color purity from grey or dull (0) to color vividness (100).

### 4.3. Total Soluble Solids (TSS) and Firmness

The TSS was detected using a pocket refractometer (PAL-1, Atago, Japan), and the results were presented as °Brix. Firmness was measured using a fruit sclerometer (WDGY-4, Beijing, China) with a probe 3.5 mm in diameter and a penetration depth of 10 mm and expressed as Newton (N). Measurements were made around the equator zone of each strawberry twice.

### 4.4. Soluble Sugar, Titratable acid (TA), and Ascorbic Acid (AsA)

The determination of soluble sugar was performed using the anthrone colorimetric method [36]. Approximately 0.2 g of fruit was extracted using distilled water. The 250 μL of the extract was added to 750 μL distilled water, 250 μL anthrone-ethyl acetate (1 g anthrone dissolved in 50 mL ethyl acetate), and 2.5 mL concentrated sulfuric acid. The mixture was placed in a water bath at 100 °C for 1 min and subsequently cooled to room temperature. The optical density of the mixture was read at 620 nm, and the content of soluble sugar was calculated by a sucrose standard curve. The titratable acidity (TA) was determined by repeated titrations with 0.1 M NaOH to a faint pink and expressed as percentage citric acid equivalents. The AsA measurement was carried out as the process described by Luo, et al. [20], the results of which were expressed as mg of AsA per 100 g of fresh weight.

### 4.5. Total Flavonoid, Phenolic, and Anthocyanin Content

Total flavonoids and phenolics were articulated by the aluminum chloride colorimetric method and Folin–Ciocalteu method, respectively, which had been described previously by Zhang, et al. [25]. A certain weight of fruit sample was mixed with 80% acetone, which turned into a clear solution of samples by centrifugation after 1 h incubation at room temperature. The absorbance of the solution for measuring total flavonoids was read at 415 nm, while the optical density of the mixture for determining total phenolics was detected at 650 nm. The calibration curves were respectively plotted using standard quercetin and gallic acid. The content of total flavonoids and phenolics were presented as milligram quercetin equivalent per kilogram of fresh weight and gram gallic acid equivalents per kilogram of a sample on a fresh weight basis, individually. The determination of total anthocyanins was conducted using the pH differential method of Zhang, et al. [25]. Two buffer solutions at different pH were used, which concluded potassium chloride buffer (0.025 M KCl) with a pH of 1.0 and sodium acetate buffer (0.4 M CH3COONa) at pH 4.5. The extract of samples was diluted with pH 1.0 buffer and pH 4.5 buffer, respectively, and then incubated for 20 min at room temperature. The absorbance was measured at 496 and 700 nm. The content of total anthocyanins was displayed as μg pelargonidin 3-glucoside equivalent kg^−1^ FW. 

### 4.6. IAA and ABA 

The endogenous ABA and IAA content were assayed by the ABA and IAA determination kit (Shanghai Enzyme-linked Biotechnology Co., Ltd., Shanghai, China), respectively. The sample (0.1 g) was extracted with 0.9 mL of PBS and centrifuged at 3000 rpm for 20 min. The supernatant was used for the ABA and IAA determination according to the manufacturer’s protocol. The optical density of the mixture was monitored at 450 nm. The IAA level was displayed as μmol of Auxin per liter of sample solution. The ABA level was expressed as ng of Abscisic acid per milliliter of sample solution.

### 4.7. Superoxide Anion, Hydrogen Peroxide, and Malondialdehyde

Superoxide anion (O_2_^−^), hydrogen peroxide (H_2_O_2_), and malondialdehyde (MDA) were measured according to the method described by Yang, et al. [37]. A total of 0.5 g fruit sample was homogenized in 3 mL ice-cold phosphate buffer (50 mM, pH 7.8) containing 1% (*w*/*v*) of polyvinylpolypyrrolidone (PVP). The homogenate was centrifuged at 12,000× *g* for 10 min at 4 °C. The upper phase (0.5 mL) was mixed with 10 mmol L^−1^ hydroxylamine hydrochloride (0.5 mL) and incubated at 25 °C for 30 min. After that, the mixture was added to 1 mL of 17 mM p-aminobenzenesulfonic acid and 1 mL of 7 mM α-naphthylamine and kept for a further 30 min at 25 °C. The absorbance was immediately recorded at 530 nm. A standard curve with sodium nitrite was employed to qualify the production rate of O_2_^−^, which was expressed as mmol min^−1^ kg^−1^.

Fruit sample (0.5 g) was extracted by 3 mL pre-cooled acetone and subsequently centrifugated at 12,000× *g* for 10 min at 4 °C. A volume of 1 mL of the resulting supernatant was added to 0.1 mL of 10% (*v*/*v*) TiCl_4_–HCl and 0.2 mL of concentrated ammonia and reacted for 5 min. The sediment after centrifugation at 12,000× *g* for 15 min at 4 °C was resolved by 3 mL of 2 M H_2_SO_4_. The optical density was read at 412 nm. A standard curve with hydrogen peroxide was used to calculate the H_2_O_2_ content, which was expressed as mmol kg^−1^.

Fruit sample (0.5 g) was homogenized with 3 mL of 100 g L ^−1^ pre-cooled trichloroacetic acid. After centrifugation at 12,000× *g* for 10 min at 4 °C, the supernatant (1 mL) was mixed with 0.67% 2-thiobarbituric acid (3 mL). The mixture was placed in the boiling water for 10 min and then immediately cooled on ice. The absorbance was spectrophotometrically measured at 450 nm, 532 nm, and 600 nm after centrifugation at 12,000× *g* for 10 min at 4 °C. The MDA content was expressed as μmol kg^−1^ of fruit fresh weight basis.

### 4.8. FRAP and DPPH

The total antioxidant activity was presented as ferric reducing antioxidant power (FRAP) and 1,1-Diphenyl-2-picryl-hydroxyl (DPPH). The detailed determination methods of FRAP and DPPH were described by Zhang, et al. [25]. The assay of FRAP was as follows. The fruit extract was blended with a fresh working FRAP reagent. The mixture was incubated at 37 °C for 30 min. After that, the absorption of the mixed solution was recorded at 593 nm. The scavenging effect was expressed as mmol kg^−1^ fresh weight. Alongside, the fruit extract was mixed with 60 μM DPPH and then the mixture was immediately placed in the dark for about 30 min at room temperature. The absorbance of the blend was taken at 517 nm using the spectrophotometer. The scavenging activity was evaluated by the inhibition percentage of DPPH.

### 4.9. RNA Extraction, cDNA Synthesis, and Quantitative Real-Time PCR (qRT-PCR)

The total RNA was extracted using the improved CTAB method [38]. The first strand of cDNA was synthesized using the PrimeScriptTM RT reagent Kit with gDNA Eraser (Takara, Dalian, China). All qRT-PCRs were conducted on the CFX96 real-time PCR system (Bio-Rad, CA, USA) using SYBR Premix (Takara, Japan). The relative gene expression levels were calculated according to the 2^−ΔΔCT^ method. Primers used for qRT-PCR are listed in Table S1.

### 4.10. Statistical Analysis

All experiments were conducted in a completely randomized design (CRD) with three replicates. The data were exhibited as mean ± standard error (SE), analyzed using IBM SPSS Statistics 23.0 in one-way analysis of (ANOVA). To test the differences between treatments, Duncan’s multiple range test was performed at the significance level of *p* ≤ 0.05. A principal components analysis (PCA) was carried out using Origin 2021 software (OriginLab Corporation, Northampton, MA, USA).

## 5. Conclusions

Taken together, both light quality and sucrose can regulate to some extent the physiological characteristics during detached strawberry ripening, but not in a simple synergistic way. Specifically, red light or blue light in combination with 100 mM sucrose may promote detached ripening via the positive regulation of abscisic acid signaling and the negative regulation of auxin signaling; however, a detailed investigation is needed.

## Data Availability

Not applicable.

## Supplementary Materials

The following supporting information can be downloaded at https://www.mdpi.com/article/10.3390/ijms24065681/s1.
