# Supplementary material for: Light Quality and Sucrose-Regulated Detached Ripening of Strawberry with Possible Involvement of Abscisic Acid and Auxin Signaling
Source: Int J Mol Sci. 2023 Mar 16;24(6):5681. doi: 10.3390/ijms24065681 (PMC10058270; doi:10.3390/ijms24065681)
Supplement: Supplementary file 1 [file ijms-24-05681-s001.zip › ijms-2229404-supplementary.pdf]

Table S1. Primers used for qRT-RT

Gene	Forward primers (5'-3')	Reverse primers (5'-3')
ABI4	TACGACCGAGCCGCTATAAT	CGTAAGGATGAGCCGAAGAG
SnRK2.6	GCTACACTCGCAACCAAAATC	ACCCCACAAGACCAGACATC
AUX/IAA11	TGGTGGTCAGGAGCATGATA	TTAGCCTCTTCACGGAACTAAGA
ARF6	AGTTTGTAAATAGTGTGTGGTGCAT	CTGCATTGGGACAGACTTCAG
ACTIN	TTCACGAGACCACCTATAACTC	GCTCATCCTATCAGCGATT
